# Spatial-Temporal resolution implementation of cloud-aerosols data through satellite cross-correlation

**DOI:** 10.1016/j.mex.2024.102547

**Published:** 2024-01-04

**Authors:** Francesca Manenti, Stefano Cavazzani, Chiara Bertolin, Sergio Ortolani, Pietro Fiorentin

**Affiliations:** a Department of Physics and Astronomy, University of Padua, Padua, Italy; b Department of Mechanical and Industrial Engineering, Norwegian University of Science and Technology, Trondheim, Norway; c Department of Industrial Engineering, University of Padua, Padua, Italy; d INAF, Osservatorio Astronomico di Padova, Padua, Italy

**Keywords:** Site testing, AOD, Atmospheric effects, Methods: statistical analysis, *Spatial-Temporal Implementation Algorithm (STIA)*

## Abstract

The Moderate Resolution Imaging Spectroradiometer (MODIS) instrument aboard Terra and Aqua satellites provides measurements of several atmospheric parameters.

This paper focuses on the cloud fraction data representing the number of cloudy pixels divided by the total number of pixels, and available through 1° x 1° grids spatial resolution with daily or monthly temporal resolution. The aim of the study is to propose a novel method called The Spatial-Temporal Implementation Algorithm (STIA) for analysing satellite daily 1° x 1°grid cloud fraction average values for

• Comparing two datasets retrieved by MODIS aboard Aqua and Terra satellites to obtain information on the cloud formation in the afternoon and morning, respectively, thus enhancing the temporal resolution.
• Comparing the actual parameter with others retrieved by instruments aboard of different satellites characterized by a better resolution. As an example of STIA application, this study uses the Aerosol Optical Depth (AOD) collected by the Ozone Monitoring Instrument (OMI) on board of Aura satellite for comparison with MODIS instrument to achieve and enhanced spatial resolution of the grid-cell.

## Graphical abstract

**Figure:**
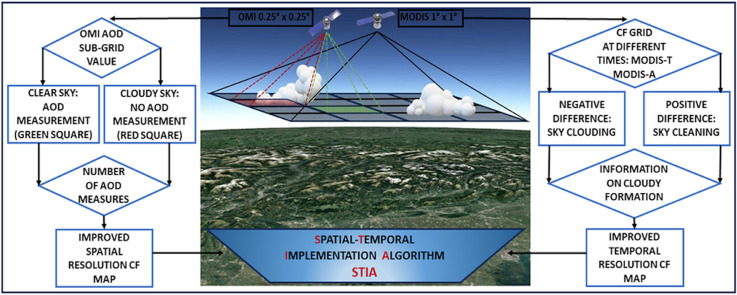

Specifications table

**Table:** 

Subject area:	Environmental Science
More specific subject area:	*Study of cloud fraction (CF) and aerosol optical depth (AOD)*
Name of your method:	*Spatial-Temporal Implementation Algorithm (STIA)*
Name and reference of original method:	*N.A.*
Resource availability:	Data can be downloaded from https://giovanni.gsfc.nasa.gov/giovanni/

## Method details

Satellite data provides an important resource for low-cost, long-term analyses of environmental parameters over the entire Earth.

In this study, a novel method to validate spatial-temporal coherence between satellite grid data and their projections at ground is proposed named STIA - Spatial-Temporal Implementation Algorithm.

To explain the developed method and test the enhanced temporal resolution, cloud fraction (CF) data stored in the following products: MOD08_D3 v6.1 [1] (MODIS-Terra) and MYD08_D3 v6.1 [2] (MODIS-Aqua) have been used. The MODIS Atmosphere Daily Global Product is stored as grid cells of 1° × 1°, which means the output grid has 360 pixels in width and 180 pixels in height. In this work, free access and almost real time data (i.e., make available with a 1-day delay) in .csv format are preferred.

Data can be downloaded from the Nasa's website Giovanni [3] https://giovanni.gsfc.nasa.gov/giovanni/. The spatial resolution of the data accomplishes with the products available in this website i.e. level 3 (L3) products that are averaged global gridded products, screened for bad data points. Although not of level 2 (L2) i.e., daily binned global products derived from the L1B product, however they allow a homogeneous analysis of the entire globe.

**Fig. 1:**
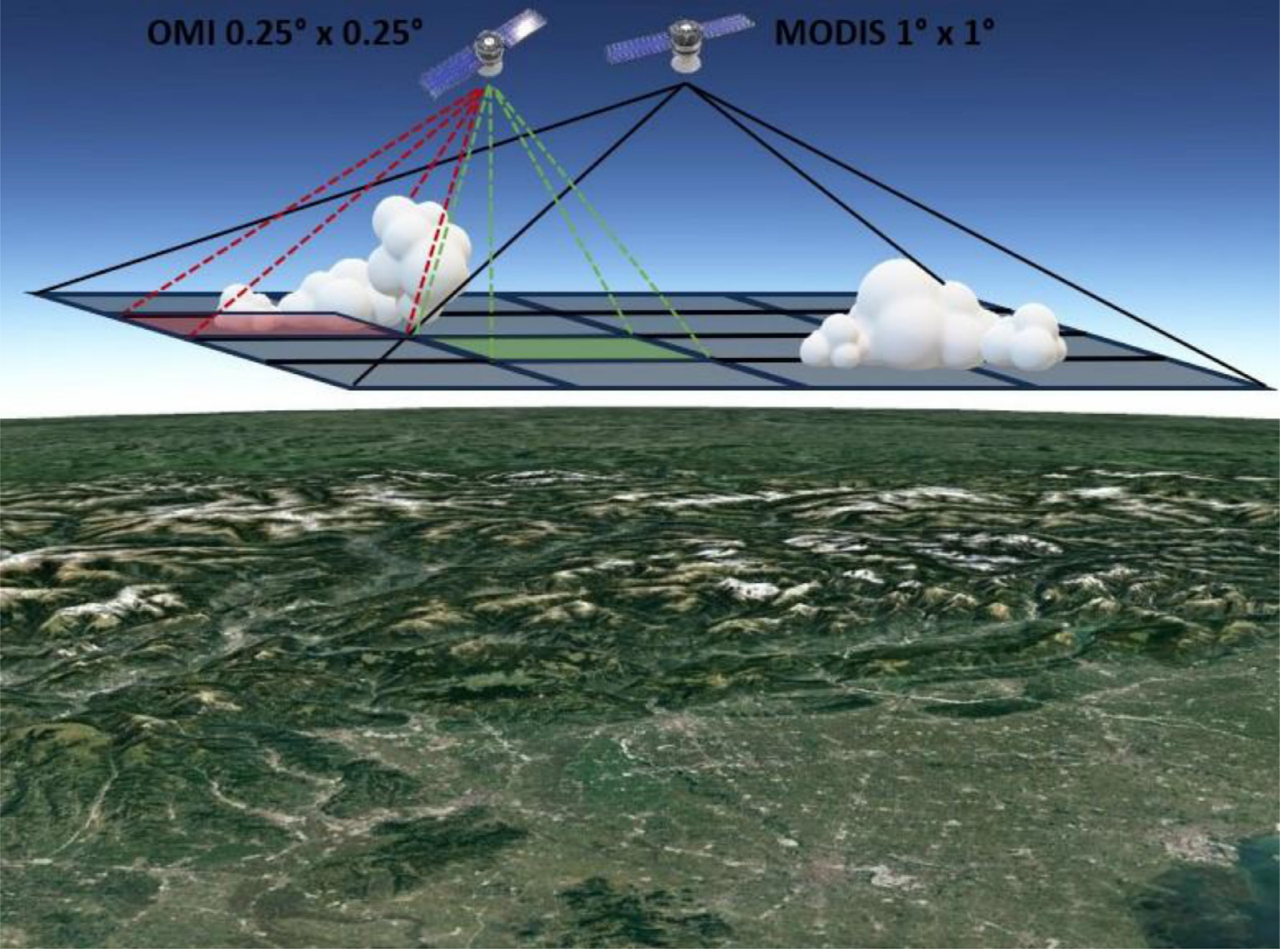
3D-scale schematic reconstruction of STIA operation. The two instruments OMI (onboard of Aura) and MODIS (onboard of Terra and Aqua) are represented with their respective spatial resolutions at ground. In the case of partial CF, the OMI data from AOD increases the spatial resolution of MODIS. The green square indicates the clear sub-matrix where OMI measures the AOD; while the red square indicates the cloudy submatrix where the AOD is not measured.

In particular, the sample region used to present the STIA method is the area bounded by the points of coordinates 11E,45 N,12E,46 N (vertices of the 1° × 1° square in Fig. 1), while the analysed temporal period is of 18 years from 01/01/2005 to the 31/12/2022. Notwithstanding, the method is replicable for all the cloud fraction datasets available in any cell of 1° × 1° over any selected interval of time (e.g., since 2000 for MODIS-Terra and 2002 for MODIS-Aqua). The original temporal resolution of MODIS-Terra and MODIS-Aqua data is daily.

While the timeseries of the aerosols optical depth (AOD) measured by the Ozone Monitoring Instrument (OMI) aboard of Aura satellite are retrieved by the OMAEROe [4] i.e., a Level-3 Aura/OMI Global Aerosol Data Product and are used to test the increased STIA spatial resolution thanks the cross-correlation between MODIS and OMI going from original 1°x1° matrices to enhanced 0.25° × 0.25° spatial resolution sub-matrices at 483.5 nm. The test area is schematized in Fig. 1. The MODIS 1° × 1° matrix is highlighted as outer black matrix in Fig. 1, while the 16 OMI submatrices with a 0.25° × 0.25° resolution each are highlighted in red and green with dashed lines in Fig. 1. They cover the sites of the city of Padua (45°24′ N, 11°53′ E, 18 m a.s.l. in Italy) and of the town of Asiago, a mountain area where the Astrophysical Observatory of the Padua University is located (45° 51′ N, 11° 34′ E 1366 m a.s.l. in Italy).

Specifically, the work, using CF and AOD time series, explicitly strengthen spatial and temporal resolution and implicitly analyses aerosol-cloud interactions.

The MODIS cloud observation bands with the related weighting functions can overestimate the CF, especially in the presence of seas, lakes and rivers in the considered matrix [5]. MODIS data are provided at various spatial resolutions (1-pixel maximum resolution of 1.0 km). However, increasing the spatial resolution leads to a decrease in the signal-to-noise ratio. Some climate models therefore use 1°x1° matrices (approximately 111 km × 111 km) to enhance the signal-to-noise ratio and reduce statistical uncertainty.

The OMI tool, however, has discontinuous data collection due to the presence of clouds as shown in Table 1 in terms of N of measurements for sub-matrices.

**Table 1:** Summary of STIA results divided into the four seasons. The top panel reports the CFAD and CFAN results measured by MODIS-Terra and MODIS-Aqua (in%). The number of AOD data (in N°) detected by OMI over the reference period (2005 to 2022) for each of the 16 sub-matrices is reported in the bottom panel.

	Winter (%)	Spring (%)	Summer (%)	Autumn (%)
CFAD_Aqua_	69.3	64.3	53.1	65.9
CFAN_Aqua_	65.7	65.3	62.5	65.3
CFAD_Terra_	68.4	60.4	48.1	63.1
CFAN_Terra_	64.2	67.9	69.3	66.5

	$N_{AOD,S}^{MATRIX}$			
Grid	Winter (in N°)	Spring (in N°)	Summer (in N°)	Autumn (in N°)

1	233	299	288	280
2	233	300	267	257
3	216	323	285	269
4	195	327	301	246
5	240	254	247	265
6	229	283	234	255
7	223	302	246	266
8	194	333	271	251
9	132	140	141	123
10	150	189	181	177
11	198	255	223	226
12	214	271	257	242
13	79	39	49	38
14	91	56	57	46
15	112	90	100	63
16	138	111	139	83
$N_{AOD,T}^{MATRIX}$	1623	1646	1644	1636

Therefore, the combination of the two time series reduces the limitations of the instruments taken individually. Finally, the aerosol-cloud interaction intrinsically analyzed by STIA have important and multidisciplinary scientific implications, also in relation to the Earth's climate and its changes, as demonstrated by numerous publications [6,7,8,9,10].

Combined CF and AOD data can also provide insights into the role of aerosols as cloud condensation nuclei (CCNs) [11,12].

The relative simplicity and versatility of STIA makes it exportable to datasets of greater spatial and temporal resolution, with high potential of exploiting the aerosol-cloud interaction in different climate zones globally.

**Fig. 2:**
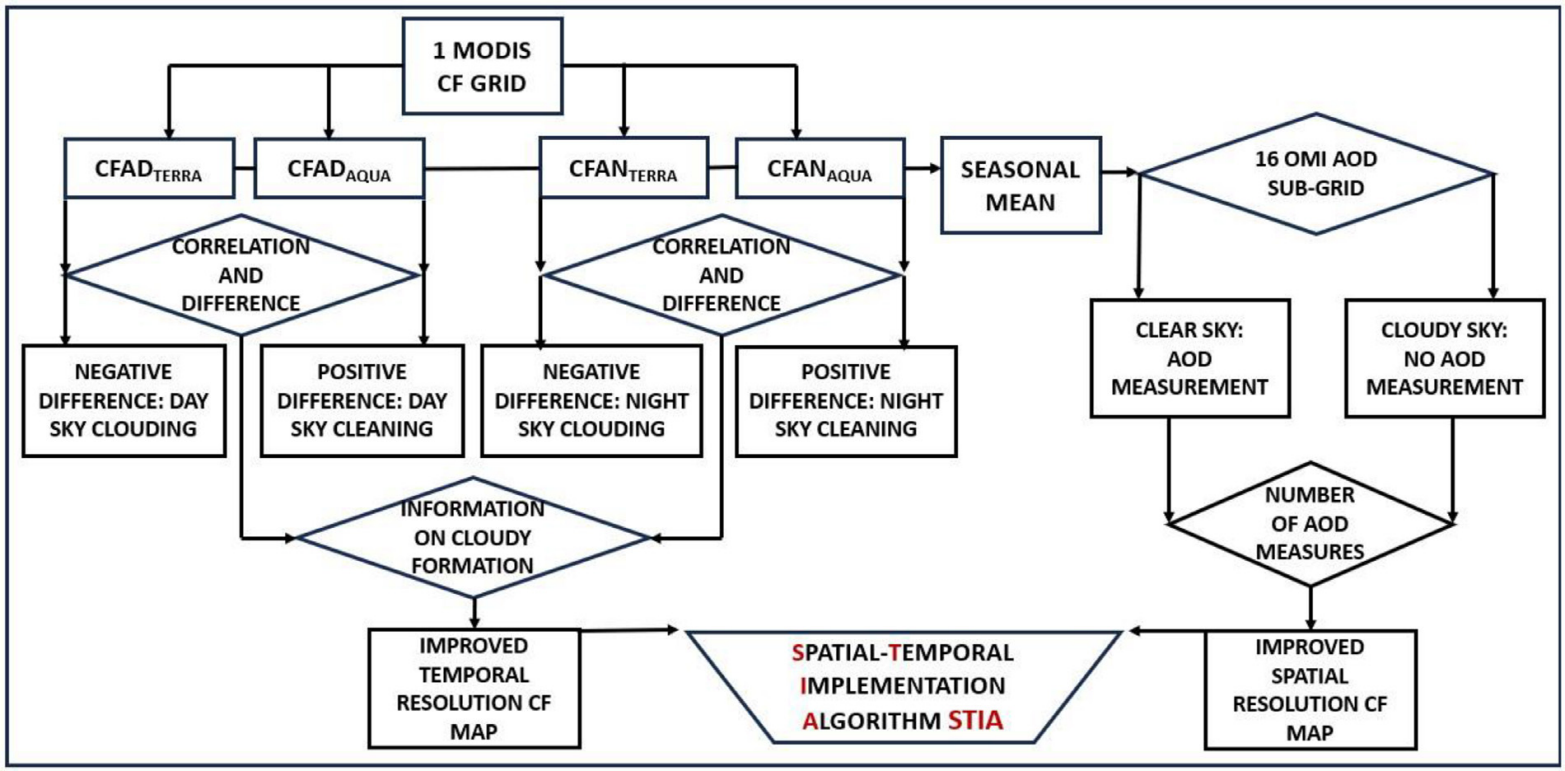
Main steps in the STIA method operation with the respective timeseries used to explain and test it and the results obtained.

**Fig. 3:**
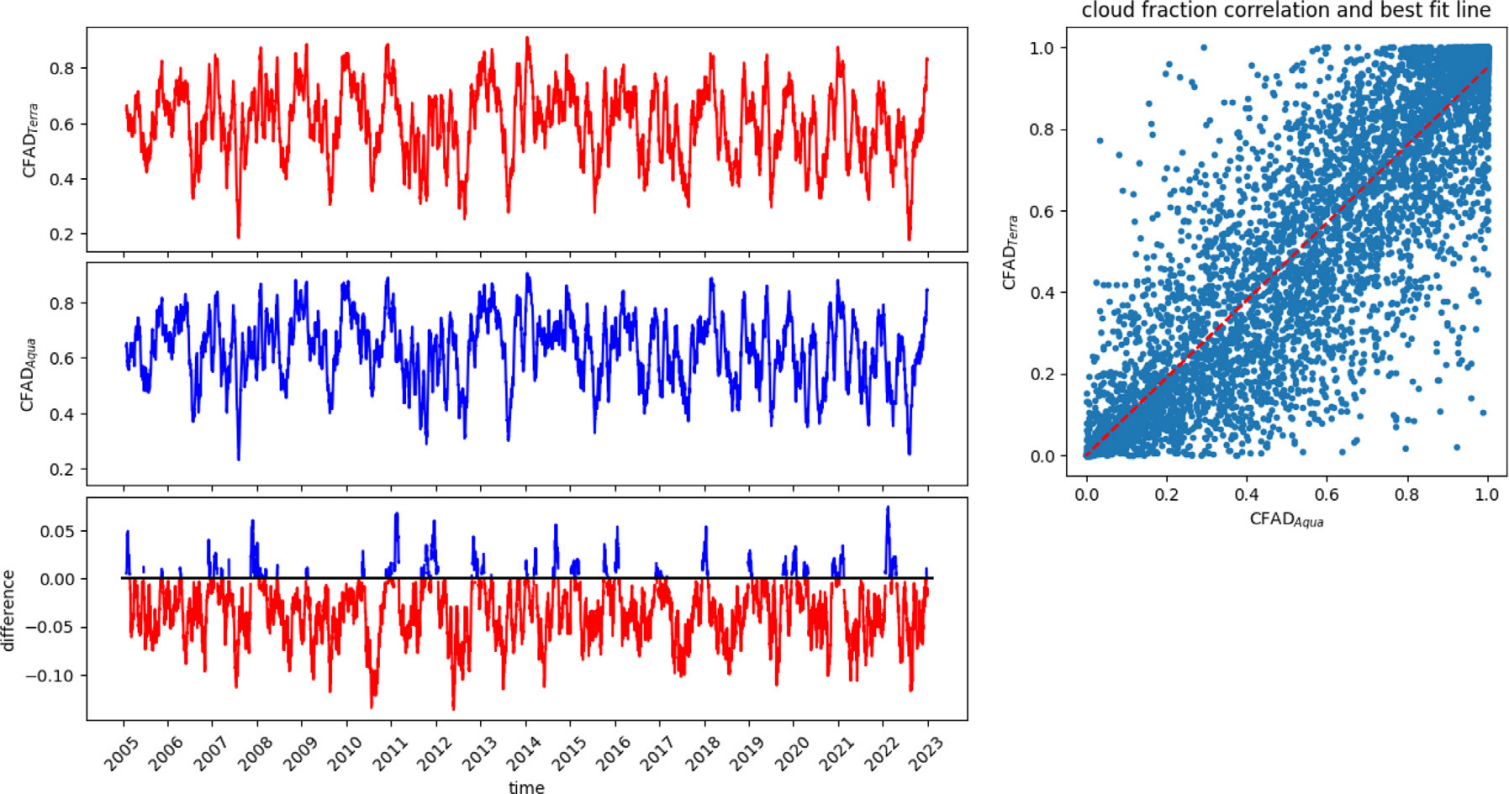
Long-term trend of CF in the 1°x1° MODIS matrix from 2005 to 2022. Comparison between 30-day moving average CFAD_Terra_ (top panel) CFAD_Aqua_ (central panel) with the respective difference CFAD_Terra_ - CFAD_Aqua_ (bottom panel). The red difference line indicates an increase in clouds between 10:30 a.m. and 1:30 p.m. (morning cloud formation), while the blue line of difference is a decrease in clouds (evening cloud formation). The top right panel shows the Pearson correlation between the two timeseries (0.90).

The Spatial-Temporal Implementation Algorithm (STIA) goes through the following steps (Fig. 2):

1. Daily analyses of CF at day (CFAD) and CF at night (CFAN) from MODIS-Terra and MODIS-Aqua are retrieved. Terra is the first EOS (Earth Observing System) platform and provides global data on the state of the atmosphere, land, and oceans, as well as their interactions with solar radiation. It has a near polar sun-synchronous orbit that passes over the equator at 10:30 a.m. (south to north) and 10:30 p.m. (north to south) in local solar time (altitude 705 km, inclination 98.1° and 98.88 min period). Aqua is a near polar sun-synchronous orbit satellite (altitude 705 km, inclination 98.2° and 98.8 min period). It passes over the equator at 1:30 p.m. (south to north) and 1:30 a.m. (north to south). Besides Terra and Aqua, this work also uses data from the polar Aura satellite: it has an orbit with an inclination of 98.22° and a 98.83 min period, with a perigee of 708 km and an apogee of 710 km. The relative temporal difference between the Terra-Aqua timeseries quantifies the cloud formation phenomenon. The four daily data subtracted from each other sample the cloud formation by intrinsically implementing the time resolution of the used polar satellites. A negative difference between 10:30 a.m. (i.e., Terra ascending observation) and 1:30 p.m. (i.e., Aqua ascending observation) detects a cloud formation process during the morning, while between 10:30 p.m. (i.e., Terra descending observation) and 1:30 a.m. (i.e., Aqua descending observation) an evening cloud formation process. Conversely, positive differences shift the cloud formation phenomenon in the afternoon or at night (Figs. 3 and 4). The Pearson correlation between the Terra-Aqua satellites shown in the right panel of Figs. 3 and 4 provides notice on the algorithm short-term prediction capacity.

**Fig. 4:**
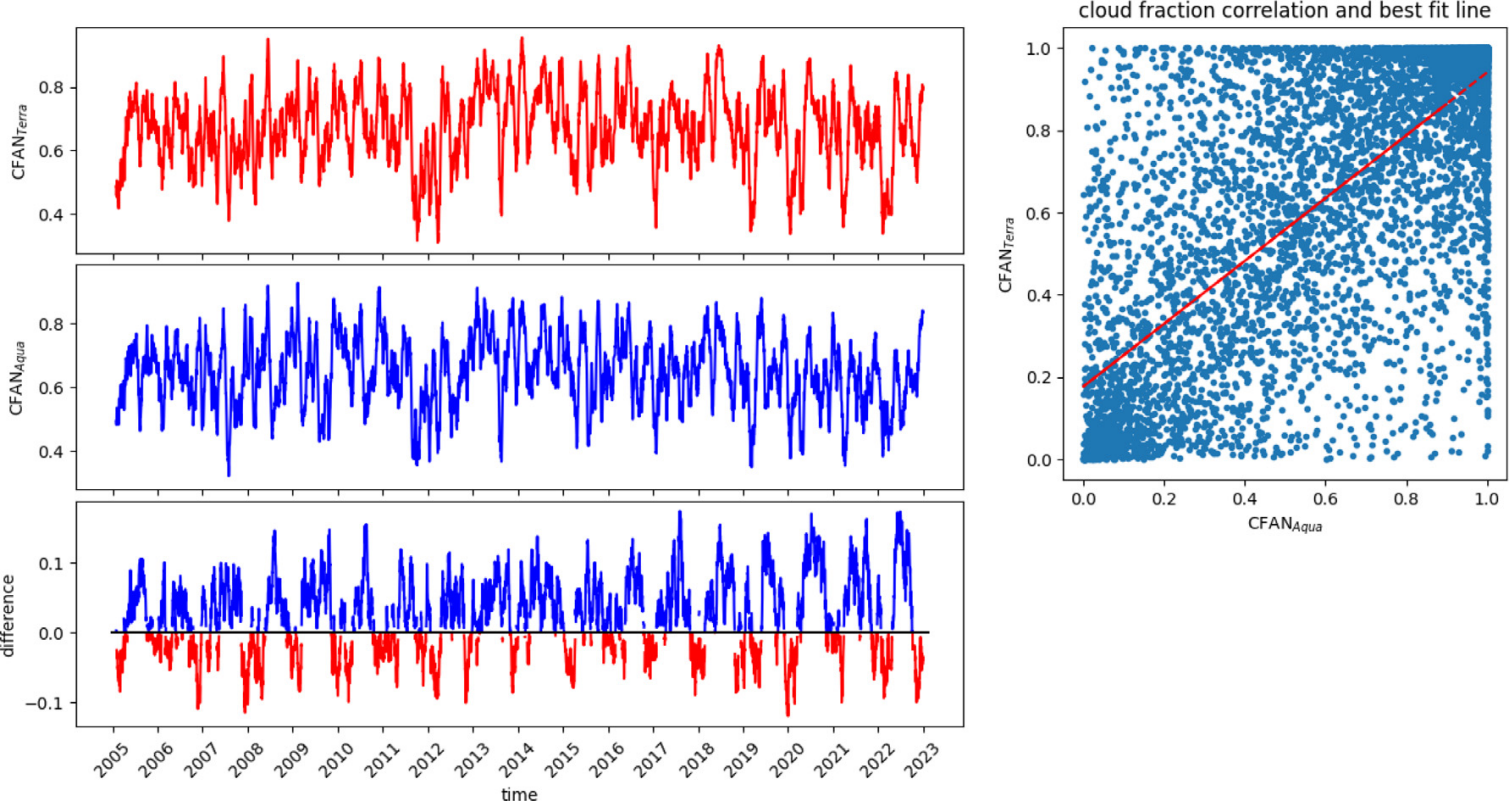
Long-term trend of CF in the 1°x1° MODIS matrix from 2005 to 2022. Comparison between 30-day moving average CFAN_Terra_ (top panel) CFAN_Aqua_ (central panel) with the respective difference CFAN_Terra_ - CFAN_Aqua_ (bottom panel). The red difference line indicates an increase in clouds between 10:30 p.m. and 1:30 a.m. (afternoon cloud formation), while the blue line of difference is a decrease in clouds (night cloud formation). The top right panel shows the Pearson correlation between the two timeseries (0.77).
2. The AURA Ozone Monitoring Instrument (OMI) uses imaging to observe back scattered solar radiation in the visible and ultraviolet. Earth is seen in the wavelength 270 to 500 nm range along the satellite track, with an area large enough to provide global coverage. The 16 OMI 0.25° × 0.25° (corresponding to an area of approximately 28 km × 28 km) sub-matrices contained in the MODIS 1.00° × 1.00° grid (corresponding to 111 km × 111 km) are used by STIA with a dual function: a direct AOD measurement function and an indirect cloud spatial sampling measurement. The AOD is measured only under clear sky conditions (green square sub-matrix in Fig. 1). While in presence of clouds, the AOD is not measured by the satellite (red square sub-matrix in Fig. 1). The number of measurements of a submatrix intrinsically detects the percentage of CFAD within the 1° × 1° matrix, thus increasing the spatial resolution of the original MODIS CF timeseries.
  (1) $$STIA_{CF,S}^{MATRIX}=\bar{CFAD}\cdot \left(1-\frac{N_{AOD,M}^{MATRIX}}{N_{AOD,T}^{MATRIX}}\right)$$
  Eq. (1) defines the operation of STIA and provides the seasonal cloud cover estimation of the sub-matrix, where $\bar{CFAD}$ is the average of CFAD_Terra_ and CFAD_Aqua_. $N_{AOD,S}^{MATRIX}$ is the number of OMI seasonal measurements of the sub-matrix and $N_{AOD,T}^{MATRIX}$ is the total number of measurements.
3. Under the assumptions in point 2 the number of OMI measurements in relation to the total number of satellite data intrinsically measures the CFAD of the sub-matrix. STIA spatially outlines the cloud formation process highlighting the seasonal percentages of CFAD with a resolution of 0.25° × 0.25°

Fig. 2 summarize the steps of the algorithm that are going to be described in the next section.

## Data analysis

MODIS and OMI tools are widely used for site testing campaigns in relation to CF and AOD [5,13,14]. Time series from 2005 to 2022 MODIS-Terra, MODIS-Aqua and OMI can therefore be used for data analysis to test the STIA method.

The plotted quantities (Figs. 3, and 4) are the 30-day moving average of the original CF data calculated with the function *DataFrame.rolling* of the Pandas package. The correlation coefficients between the time series of CFAD_Terra_ and CFAD_Aqua_, and between CFAN_Terra_ and CFAN_Aqua_ are 0.90 and 0.77 respectively (using the pre-defined function *np.corrcoef* of the NumPy module of Python).

The data analysis of Fig. 3 highlights the daily seasonal phenomena of cloud formation. During the day in spring, summer, and autumn there is a prevalence of cloud formation between 10:30 a.m. and 1:30 p.m., while in winter a clearing of the sky. From the figure a seasonal trend can also be recognized as the daily cloud coverage evolution process occurs in the spring and summer seasons, while the clearing process occurs in winter (blue peaks in Fig. 3, bottom panel).

Overall, there is no evidence, in the studied interval, of a long-term trend, except a possible minor decrease in the 2019–2022 years. Notwithstanding over the analyzed datasets there are wide seasonal and yearly cloud coverage variations, for example a deep minimum in 2012, followed by a higher value in 2013.

**Fig. 5:**
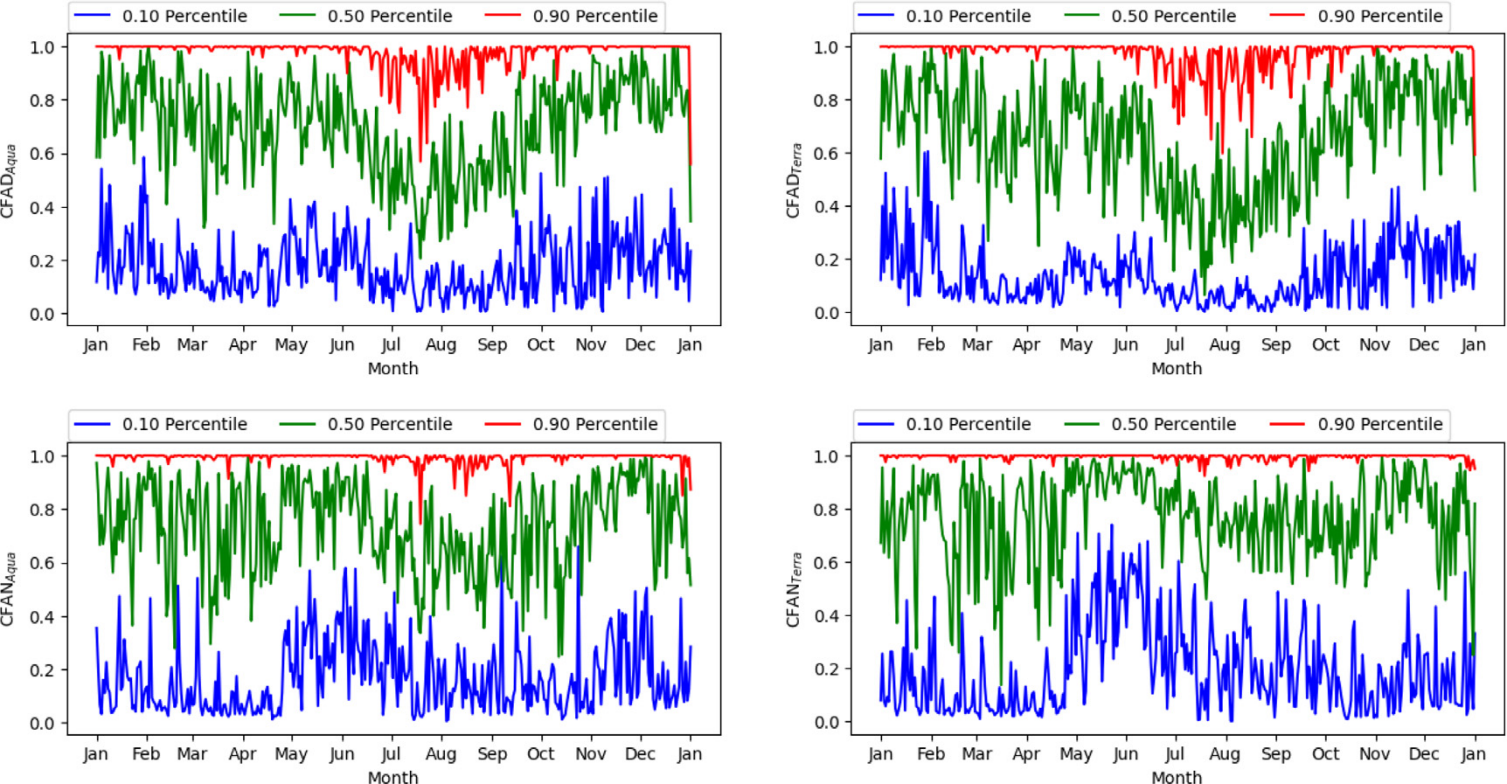
Seasonal variability of CFAD_Terra_,CFAD_Aqua,_ CFAN_Terra_, CFAN_Aqua_ showed by different percentiles, in blue the 10th percentile; in green the 50th percentile and in red the 90th percentile 2005 to 2022.

**Fig. 6:**
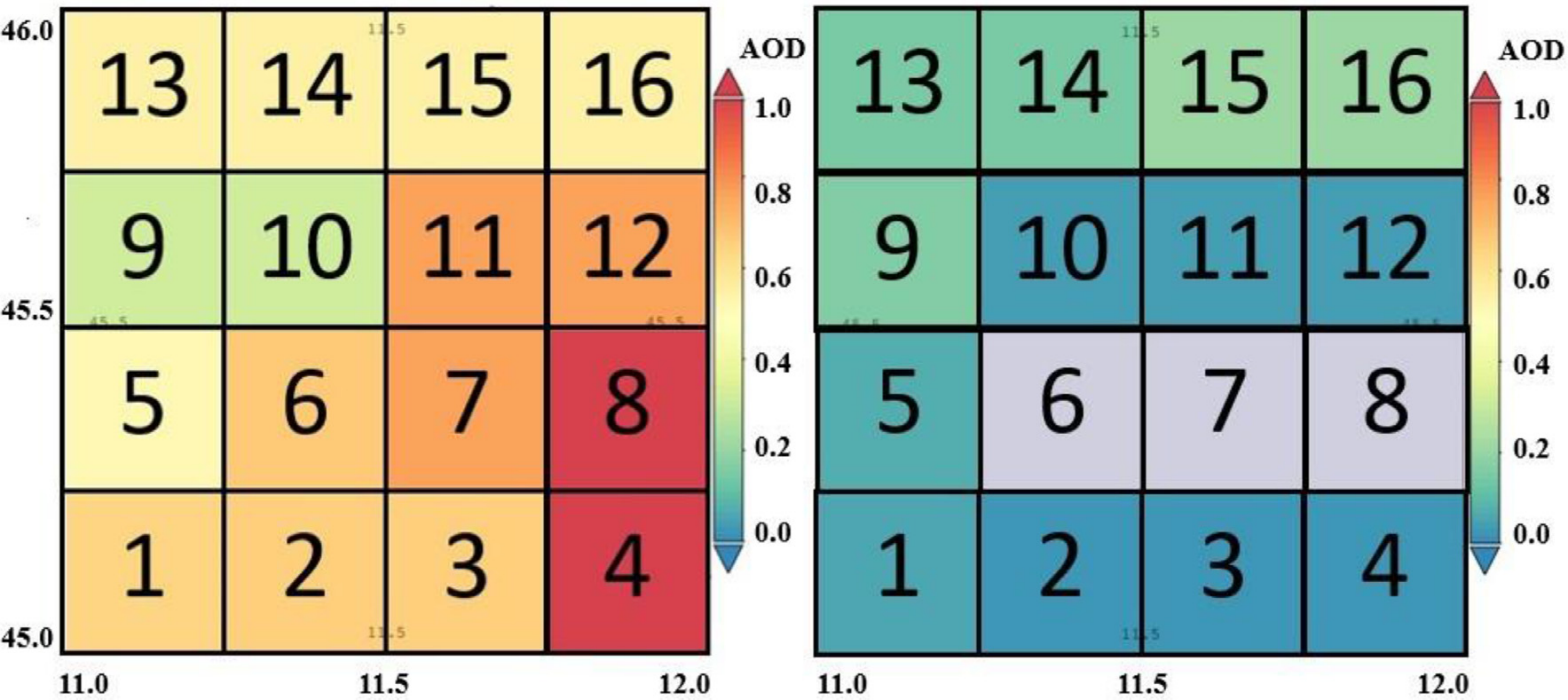
Example of AOD measurements by OMI in the 16 sub-matrices. The left panel shows a completely clear day, while the right panel shows a partly cloudy day. On the right, STIA method classifies submatrices 6, 7 and 8 as cloudy where the AOD cannot be measured.

Fig. 4 displays, the timeseries at night. In spring, summer, and autumn, in the second part of the night, between 10:30 p.m. and 1:30 a.m., the sky gets preferentially clear, while it gets cloudy, during the first part of the night, in winter (Fig. 4). Ground and satellite data confirm this result [15,16,17]. Seasonal variability are highlighted in Fig. 5, where the values of the 10th (blue line), 50th (green value) and 90th (red line) percentile are represented. The 10th and 90th percentiles represent the typical year with the lowest and highest CF, respectively.Fig. 6 outlines the operation of STIA with two examples of AOD map measured by OMI using OMAEROe [4] i.e., a Level-3 Aura/OMI Global Aerosol Data Product that selects the best aerosol value from the Level2G good quality data that are reported in each sub-grid. The key factor is that in presence of cloud cover OMI doesn't measure the AOD value since different tests are applied to exclude cloudy scenes from the retrieval [18].

The left panel of Fig. 6 outlines an example of a clear day in which AOD can be detected in all the OMI sub-matrices; while the right panel shows an example of a cloudy day when OMI does not measure AOD in sub-matrices 6, 7, 8 (grey color, right panel in Fig. 6) intrinsically detecting cloud cover over the respective geographic area.

It is worthy to mention that during data analysis OMI AOD values larger than 1 have been ignored because they were outliers due to the Aura satellite inclined observation's angle.

Tables 1 and 2 summarize the overall STIA results after the completion of all the steps reported in Fig. 2. Seasonal CFAD and CFAN measured (in%) by MODIS-Terra and MODIS-Aqua (see steps in Fig. 2) are reported in the top panel of Table 1. These numbers have been obtained by using statistical functions of the NumPy module of Python. Then the number of OMI AOD measurements, for each of the 16 sub-grids (Fig. 2 and Table 1), have been calculated. Considering the total number of values that should have been detected in absence of cloud cover over each single grid is possible to calculate the detection rate (reported in number of values) used by STIA. AOD mean values ($\bar{AOD_{S}}$) have also been calculated (Table 2). The STIA results in Table 2 give an important estimate of the real cloud fraction (i.e., CF in%) with an improved spatial resolution of 0.25° × 0.25° respect to the original one of 1.0° × 1.0° provided by MODIS.

**Table 2:** Average AOD values (pure number) measured per each of the 16 OMI sub-matrices in relation to the calculated $STIA_{CF,S}^{MATRIX}$ percentage (%) obtained from Eq. (1) 2005 to 2022.

	$\bar{AOD_{S}}$	$STIA_{CF,S}^{MATRIX}\;\left(\%\right)$	$\bar{AOD_{S}}$	$STIA_{CF,S}^{MATRIX}\;\left(\%\right)$	$\bar{AOD_{S}}$	$STIA_{CF,S}^{MATRIX}\;\left(\%\right)$	$\bar{AOD_{S}}$	$STIA_{CF,S}^{MATRIX}\;\left(\%\right)$
Grid	Winter		Spring		Summer		Autumn	
1	0.21	59.0	0.34	51.0	0.27	41.7	0.30	53.5
2	0.20	59.0	0.34	51.0	0.28	42.4	0.30	54.4
3	0.20	59.7	0.37	50.1	0.26	41.8	0.30	53.9
4	0.20	60.6	0.39	50.0	0.26	41.3	0.30	54.8
5	0.21	58.7	0.36	52.7	0.27	43.0	0.31	54.1
6	0.20	59.1	0.36	51.6	0.28	43.4	0.29	54.4
7	0.19	59.4	0.36	50.9	0.28	43.0	0.28	54.0
8	0.19	60.6	0.38	49.7	0.28	42.3	0.27	54.6
9	0.21	63.3	0.37	57.0	0.35	46.3	0.33	59.7
10	0.19	62.5	0.37	55.2	0.33	45.0	0.30	57.5
11	0.20	60.5	0.35	52.7	0.35	43.7	0.32	55.6
12	0.20	59.8	0.38	52.1	0.35	42.7	0.31	55.0
13	0.28	65.5	0.47	60.9	0.40	49.1	0.29	63.0
14	0.25	65.0	0.43	60.2	0.42	48.8	0.32	62.7
15	0.26	64.1	0.36	58.9	0.41	47.5	0.36	62.0
16	0.22	63.0	0.33	58.1	0.36	46.3	0.31	61.2

The code used to obtain the values of Table 1 and 2 as well as the one used to build Fig. 3, Fig. 4, Fig. 5 is added as supplementary material.

The STIA validation and the error estimation associated with the measurements is obtained by the comparison with ground data. Statistics from ground weather stations (https://www.meteoblue.com/) over the last 30 years (1991–2020) are used to this scope for both the Padua site (Matrix 8 in Fig. 6) and Asiago site (Matrix 15 in Fig. 6).

Table 3 compares the seasonal CF time calculated with STIA with that measured from the ground. The absolute bias between the two percentages provides the STIA uncertainty expressed in percentage ($\varepsilon_{\%}=\;\left|CF_{Ground}-CF_{STIA}\right|$) and in days calculating the number per each month constituting the selected season (e.g. winter $\varepsilon_{day}=\left(31+31+28\right)\cdot \frac{\varepsilon_{\%}}{100}$). We can therefore estimate an average uncertainty of less than 3.0 percent for the measured seasonal CF percentage. The described statistical method allows an increase in spatial and temporal resolution estimating also the percentage of clear skies during the days classified as covered in accordance with ground-based statistics (e.g., MODIS detects an average CFAD of 62 % for the considered area, while STIA provides an average CFAD of 52 % at Padua and 58 % at Asiago) [5].

**Table 3:** Validation and uncertainty of STIA. Comparison between the seasonal CF time from the ground (columns 2 and 6) and the value calculated with STIA (columns 3 and 7). Uncertainty in percentage (columns 4 and 8) and days (columns 5 and 9).

	Padua	Matrix 8			Asiago	Matrix 15		
	Ground CF	STIA CF	ε [%]	ε [day]	Ground CF	STIA CF	ε [%]	ε [day]
Winter	59,3	60,6	1,3	1,1	61,3	64,1	2,8	2,5
Spring	47,0	49,7	2,7	2,5	59,7	58,9	0,8	0,7
Summer	38,4	42,3	3,9	3,6	49,6	47,5	2,1	1,9
Autumn	52,0	54,6	2,6	2,3	58,0	62,0	4,0	3,7
Mean	49,2	51,8	2,6	2,4	57,1	58,1	2,4	2,2

STIA therefore provides a more reliable information, reducing the possible overestimation of the CF as measured by MODIS. In future work, the dependence of the AOD measurement on the altitude and inclination of the satellite orbit will be further analyzed for enhancing the STIA method implementation.

*Pdf file in the supplementary material section contains the Python code used to obtain the values of* Tables 1 *and* 2 *; it is reported also the one for* Fig. 3 *(same for* Fig. 4 *changing datasets) and for* Fig. 5 *.*

*Code 1: Mean of CF and N. of AOD measurements (* Table 1 *)and AOD mean value(* Table 2 *)*

*Code 2:* Fig. 3 *. CFAD_Terra_, CFAD_Aqua_ and the respective difference CFAD_Terra_ - CFAD_Aqua_ (left panels) and on the right panel correlation coefficient between CFAD_Terra_ and CFAD_Aqua_*

*Code 3:* Fig. 5 *. Seasonal variability of CFAD_Terra_,CFAD_Aqua_, CFAN_Terra_, CFAN_Aqua_ with different percentiles*

## CRediT authorship contribution statement

**Francesca Manenti:** Data curation, Formal analysis, Methodology, Software, Writing – original draft. **Stefano Cavazzani:** Conceptualization, Methodology, Software, Data curation, Validation. **Chiara Bertolin:** Investigation, Validation, Visualization, Supervision, Writing – review & editing. **Sergio Ortolani:** Supervision. **Pietro Fiorentin:** Validation.

## Declaration of Competing Interest

The authors declare that they have no known competing financial interests or personal relationships that could have appeared to influence the work reported in this paper.

## Data Availability

Data will be made available on request.

## Appendix Supplementary materials

Image, application 1
