# Supplementary material for: Spatial-Temporal resolution implementation of cloud-aerosols data through satellite cross-correlation
Source: MethodsX. 2024 Jan 4;12:102547. doi: 10.1016/j.mex.2024.102547 (PMC10825479; doi:10.1016/j.mex.2024.102547)
Supplement: Supplementary file 1 [file mmc1.pdf]

```
#code used for the results in Tab. 1 and 2
import numpy as np
import pandas as pd
import matplotlib.pyplot as plt
plt.rcParams.update({'font.size': 15})

def mesi(input,m1,m2,m3):
    return input.loc[(input.time.dt.month==m1) |
                     (input.time.dt.month==m2) | (input.time.dt.month==m3)]

a_day=pd.read_csv('aqua_day.csv',header=7,parse_dates=['time'],
                  skiprows=(1459,1460,4075,4076,4077,4078,4079,4080,4081,4082,
                            4083,6485,6486,6487,6488,6489,6490,6491,6492
                            ,6493,6494,6495,6496))

a_night=pd.read_csv('aqua_night.csv',header=7,parse_dates=['time'],
                    skiprows=(1459,1460,4075,4076,4077,4078,4079,4080,4081,4082,
                            4083,6485,6486,6487,6488,6489,6490,6491,6492
                            ,6493,6494,6495,6496))

t_day=pd.read_csv('terra_day.csv',header=7,parse_dates=['time'],
                  skiprows=(6297,6298,6299,6300,6301,6302,6303,6304,6305,6306,
                            6307,6308,6309,6310,6311,6312))

t_night=pd.read_csv('terra_night.csv',header=7,parse_dates=['time'],
                    skiprows=(6297,6298,6299,6300,6301,6302,6303,6304,6305,6306,
                            6307,6308,6309,6310,6311,6312))

#use DataFrame.drop() to delete rows in order to make the above
#time series homogeneous

aod1=pd.read_csv('1_483.csv',header=7,parse_dates=['time'])
```

```
#upload all the csv file for the 16 matrices of OMI


aod1=aod1[(aod1.OMAEROe_003_AerosolOpticalThicknessMW_483_5 < 1)
       & (aod1.OMAEROe_003_AerosolOpticalThicknessMW_483_5 != -32767)]


dataset=[a_day,a_night,t_day,t_night,aod1,aod2,aod3,aod4,aod5,aod6,aod7,
        aod8,aod9,aod10,aod11,aod12,aod13,aod14,aod15,aod16]


primavera=[]
estate=[]
autunno=[]
inverno=[]


for i in range(0,20,1):
   pr=mesi(dataset[i],3,4,5)
   es=mesi(dataset[i],6,7,8)
   au=mesi(dataset[i],9,10,11)
   inv=mesi(dataset[i],12,1,2)
   primavera.append(pr)
   estate.append(es)
   inverno.append(inv)
   autunno.append(au)
   if i>3:
      print('dati inverno',len(inverno[i]))
      print ('dati primavera',len(primavera[i]))    #n of AOD measurements
      print ('dati estate',len(estate[i]))
      print ('dati autunno',len(autunno[i]))
      print('media AOD inverno',np.mean(inverno[i]))
      print('media AOD primavera',np.mean(primavera[i]))
      print('media AOD estate',np.mean(estate[i]))        #mean of AOD
      print('media AOD autunno',np.mean(autunno[i]))
```

```
media_0=np.mean(estate[0])
media_1=np.mean(estate[1])
media_2=np.mean(estate[2])
media_3=np.mean(estate[3])     #mean of the cloud fraction

print('media:',media_0)
print('media:',media_1)
print('media:',media_2)
print('media:',media_3)




#code used for Fig. 3: CFADTerra , CFADAqua(and their correlation coefficient)
# and the respective difference CFADTerra - CFADAqua
import numpy as np
import pandas as pd
import matplotlib.pyplot as plt
import matplotlib.gridspec as gridspec

dfA=pd.read_csv('aqua_day.csv',header=7,parse_dates=['time'],
              skiprows=(1459,1460,4075,4076,4077,4078,4079,4080,4081,4082,
                        4083,6485,6486,6487,6488,6489,6490,6491,6492
                        ,6493,6494,6495,6496))
dfT=pd.read_csv('terra_day.csv',header=7,parse_dates=['time'],
               skiprows=(6297,6298,6299,6300,6301,6302,6303,6304,6305,6306,
                        6307,6308,6309,6310,6311,6312))
dfA=dfA.drop(dfA.index[[137,536,599,707,922,1449,1450,1778,2185,2448,2537,
                   2905,3149,3536,3583,3599,3849,4240,4485,5343,5695,5703,
                   5711,6098,6099,6465,6522]])
dfT=dfT.drop(dfT.index[[137,536,599,707,922,1449,1450,1778,2185,2448,2537,
                   2905,3149,3536,3583,3599,3849,4240,4485,5343,5695,5703,
                   5711,6098,6099,6465,6522]])
```

```
x=dfT["time"]
y=dfT["cf"]
xA=dfA["time"]
yA=dfA["cf"]

print(x.equals(xA))#check that the timeseries are homogeneous

media=dfA.set_index("time").rolling(30).mean()
mediaT=dfT.set_index("time").rolling(30).mean()
diff=(mediaT-media)

fig=plt.figure()
gs=gridspec.GridSpec(1,2,figure=fig,wspace=0.15,width_ratios=[2,1])
gs0=gridspec.GridSpecFromSubplotSpec(3,1,
            subplot_spec=gs[0],hspace=0.05)

ax1=fig.add_subplot(gs0[0])
ax2=fig.add_subplot(gs0[1],sharex=ax1,sharey=ax1)
ax3=fig.add_subplot(gs0[-1],sharex=ax1)
ax3.tick_params(axis='x', rotation=45)

from matplotlib.dates import YearLocator, DateFormatter
ax3.xaxis.set_major_locator(YearLocator(1))
ax3.xaxis.set_major_formatter(DateFormatter('%Y'))
gs1=gridspec.GridSpecFromSubplotSpec(3,1,subplot_spec=gs[1])
ax4=fig.add_subplot(gs1[:-1,0])

ax1.plot(x,mediaT,color='red')
ax1.set_ylabel('CFAD$_{Terra}$')
ax2.plot(xA, media,color='blue')
ax2.set_ylabel('CFAD$_{Aqua}$')
```

```
diffmagg=diff.cf.where(diff.cf.ge(0),np.nan)
diffmin=diff.cf.where(diff.cf.lt(0),np.nan)

ax3.plot(diffmagg,'blue')
ax3.plot(diffmin,'red')
ax3.axhline(y=0.0, xmin=0.04, xmax=0.96,color='black')
ax3.set_ylabel('difference')
ax3.set_xlabel('time')

#correlation

corr=np.corrcoef(yA,y)

print('matrice',corr)
covariance = np.cov(yA, y)[0][1]
st_dev = np.std(yA)
dev=np.std(y)
r=covariance/(st_dev*dev)
print('correlazione',r)
yAmedio=np.mean(yA)
ymedio=np.mean(y)
N=yA.size
sumyA=np.sum(yA)
sumy=np.sum(y)
sumyA2=np.sum(yA**2)
sumyAy=np.sum(yA*y)
delta=N*sumyA2-sumyA*sumyA
A=(sumyA2*sumy-(sumyA*sumyAy))/delta
B=(N*sumyAy-(sumyA*sumy))/delta
sigmay=((1/(N-2))*(np.sum((y-A-B*yA)**2)))**(1/2)
sigmaA=sigmay*((sumyA2/delta)**(1/2))
sigmaB=sigmay*((N/delta)**(1/2))
```

```
yy=A+B*yA
print('b',B)
print('a',A)
print(sigmay)
ax4.scatter(yA,y,marker='.')
ax4.plot(yA,yy,color='red',linestyle='--',label='best fit')
ax4.set_xlabel('CFAD$_{Aqua}$')
ax4.set_ylabel('CFAD$_{Terra}$')
ax4.set_title('cloud fraction correlation and best fit line')
plt.show()



#code for Fig. 5: Seasonal variability of CFADTerra,CFADAqua,
#CFANTerra, CFANAqua with different percentiles


import numpy as np
import pandas as pd
import matplotlib.pyplot as plt
import matplotlib.gridspec as gridspec
import seaborn as sns
import matplotlib.dates as mdates

#plt.rcParams.update({'font.size': 15})

dfA=pd.read_csv('aqua_day.csv',header=7,parse_dates=['time'],
             skiprows=(1459,1460,4075,4076,4077,4078,4079,4080,4081,4082,
                    4083,6485,6486,6487,6488,6489,6490,6491,6492
                    ,6493,6494,6495,6496))
dfT=pd.read_csv('terra_day.csv',header=7,parse_dates=['time'],
              skiprows=(6297,6298,6299,6300,6301,6302,6303,6304,6305,6306,
                    6307,6308,6309,6310,6311,6312))
```

```
dfA=dfA.drop(dfA.index[[137,536,599,707,922,1449,1450,1778,2185,2448,2537,
                    2905,3149,3536,3583,3599,3849,4240,4485,5343,5695,5703,
                    5711,6098,6099,6465,6522]])
dfT=dfT.drop(dfT.index[[137,536,599,707,922,1449,1450,1778,2185,2448,2537,
                    2905,3149,3536,3583,3599,3849,4240,4485,5343,5695,5703,
                    5711,6098,6099,6465,6522]])

dfA_N=pd.read_csv('aqua_night.csv',header=7,parse_dates=['time'],
              skiprows=(1459,1460,4075,4076,4077,4078,4079,4080,4081,4082,
                        4083,6485,6486,6487,6488,6489,6490,6491,6492
                        ,6493,6494,6495,6496))

dfT_N=pd.read_csv('terra_night.csv',header=7,parse_dates=['time'],
               skiprows=(6297,6298,6299,6300,6301,6302,6303,6304,6305,6306,
                        6307,6308,6309,6310,6311,6312))




dfA_N=dfA_N.drop(dfA_N.index[[53,78,364,535,549,598,1048,1076,
                     1094,1201,1449,1708,1831,2627,2685,2861,2962,3101,
                     3649,4063,4724,5006,5017,5700,
                     5705,5707,5715,5746,6097,6098,6287,6464,6465]])
dfT_N=dfT_N.drop(dfT_N.index[[53,78,364,535,549,598,1048,1076,
                     1094,1201,1449,1708,1831,2627,2685,2861,2962,3101,
                     3649,4063,4724,5006,5017,5700,
                     5705,5707,5715,5746,6097,6098,6287,6464,6465]])



dfT["time"]= dfT['time'].dt.strftime('%j')
data_1_T=dfT.groupby(['time']).quantile(0.10)
```

```
data_9_T=dfT.groupby(['time']).quantile(0.90)
data_5_T=dfT.groupby(['time']).quantile(0.50)

dfA["time"]= dfA['time'].dt.strftime('%j')
data_1_A=dfA.groupby(['time']).quantile(0.10)
data_9_A=dfA.groupby(['time']).quantile(0.90)
data_5_A=dfA.groupby(['time']).quantile(0.50)

dfA_N["time"]= dfA_N['time'].dt.strftime('%j')
data_1_A_N=dfA_N.groupby(['time']).quantile(0.10)
data_9_A_N=dfA_N.groupby(['time']).quantile(0.90)
data_5_A_N=dfA_N.groupby(['time']).quantile(0.50)

dfT_N["time"]= dfT_N['time'].dt.strftime('%j')
data_1_T_N=dfT_N.groupby(['time']).quantile(0.10)
data_9_T_N=dfT_N.groupby(['time']).quantile(0.90)
data_5_T_N=dfT_N.groupby(['time']).quantile(0.50)

fig,axs = plt.subplots(2,2)

data_1_T.plot(ax=axs[0,1],c='blue')
data_5_T.plot(ax=axs[0,1],c='green')
data_9_T.plot(ax=axs[0,1],c='red')
axs[0,1].set_xlabel('Month')
axs[0,1].set_ylabel('CFAD$_{Terra}$')

data_1_A.plot(ax=axs[0,0],c='blue')
data_5_A.plot(ax=axs[0,0],c='green')
data_9_A.plot(ax=axs[0,0],c='red')
axs[0,0].set_xlabel('Month')
axs[0,0].set_ylabel('CFAD$_{Aqua}$')
```

```
data_1_A_N.plot(ax=axs[1,0],c='blue')
data_5_A_N.plot(ax=axs[1,0],c='green')
data_9_A_N.plot(ax=axs[1,0],c='red')
axs[1,0].set_xlabel('Month')
axs[1,0].set_ylabel('CFAN$_{Aqua}$')

data_1_T_N.plot(ax=axs[1,1],c='blue')
data_5_T_N.plot(ax=axs[1,1],c='green')
data_9_T_N.plot(ax=axs[1,1],c='red')
axs[1,1].set_xlabel('Month')
axs[1,1].set_ylabel('CFAN$_{Terra}$')

axs[0,0].legend(['0.10 Percentile','0.50 Percentile','0.90 Percentile'],
        bbox_to_anchor=(0, 1), loc='lower left', borderaxespad=0.1, ncols=3)
axs[1,0].legend(['0.10 Percentile','0.50 Percentile','0.90 Percentile'],
        bbox_to_anchor=(0, 1), loc='lower left', borderaxespad=0.1, ncols=3)
axs[0,1].legend(['0.10 Percentile','0.50 Percentile','0.90 Percentile'],
        bbox_to_anchor=(0, 1), loc='lower left', borderaxespad=0.1, ncols=3)
axs[1,1].legend(['0.10 Percentile','0.50 Percentile','0.90 Percentile'],
        bbox_to_anchor=(0, 1), loc='lower left', borderaxespad=0.1, ncols=3)



axs[0,0].xaxis.set_major_locator(mdates.MonthLocator(interval=1) )
axs[0,0].xaxis.set_major_formatter(mdates.DateFormatter('%b'))

axs[1,1].xaxis.set_major_locator(mdates.MonthLocator(interval=1) )
axs[1,1].xaxis.set_major_formatter(mdates.DateFormatter('%b'))

axs[1,0].xaxis.set_major_locator(mdates.MonthLocator(interval=1) )
axs[1,0].xaxis.set_major_formatter(mdates.DateFormatter('%b'))

axs[0,1].xaxis.set_major_locator(mdates.MonthLocator(interval=1) )
```

```
axs[0,1].xaxis.set_major_formatter(mdates.DateFormatter('%b'))


plt.subplots_adjust(hspace=0.4)

plt.show()
```
